# Association Between Changes in Perceived Neighborhood and Cognitive Performance Within Healthy Aging in Neighborhoods of Diversity Across the Life Span (HANDLS) Black Adults

**DOI:** 10.3390/ijerph23081055

**Published:** 2026-08-14

**Authors:** Alexa C. Allan, Alyssa A. Gamaldo, Roland J. Thorpe, Jennifer D. Roberts, Michele K. Evans, Alan B. Zonderman

**Affiliations:** 1 Department of Human Development and Family Studies, The Pennsylvania State University, University Park, PA 16802, USA; 2 Institute for Engaged Aging, Department of Psychology, Clemson University, Seneca, SC 29672, USA; agamald@clemson.edu; 3 Johns Hopkins Alzheimer’s Disease Resource Center for Minority Aging Research, Bloomberg School of Public Health, Baltimore, MD 21205, USA; rthorpe@jhu.edu; 4 Department of Kinesiology, University of Maryland, College Park, MD 20742, USA; jenrob@umd.edu; 5 Laboratory of Epidemiology and Population Sciences, National Institute on Aging, Baltimore, MD 21224, USA; evansm@grc.nia.nih.gov (M.K.E.); zondermana@mail.nih.gov (A.B.Z.)

**Keywords:** cognitive performance, neighborhood quality, neighborhood perception

## Abstract

**Highlights:**

**Public health relevance—How does this work relate to a public health issue?**

- This work highlights the importance of examining changes in contextual factors (i.e., perceived neighborhood characteristics) as they relate to cognitive function.

**Public health significance—Why is this work of significance to public health?**

- As Black adults are at a disproportionate risk for diagnosis of Alzheimer’s disease and related dementias and more likely to face sociocontextual events (e.g., gentrification, cultural displacement) across the life course, the study findings highlight the role of perceived neighborhood characteristics and cognitive function.
- Changes in perceived neighborhood characteristics (e.g., positive and negative) related to various domains of cognitive functioning in opposing ways, emphasizing the need to focus on the role of changing perceived neighborhood characteristics as they relate to cognitive functioning, particularly in a sample of socioeconomically diverse mid-life-to-older Black adults.

**Public health implications—What are the key implications or messages for practitioners, policy makers and/or researchers in public health?**

- Study findings highlight new potential research directions to explore, such as the potential connection between changes in neighborhood perceptions and cognitive functioning.
- Understanding how changes in neighborhood perceptions relate to health outcomes like cognitive function is a necessary step toward understanding how these factors can shape resource availability and improve public policy.

**Abstract:**

Perceived neighborhood characteristics are related to cognitive performance. However, limited studies have examined how changes in neighborhood characteristics relate to cognitive performance, particularly among Black adults who are likely to experience changes in their residential social and physical environments. This study examined the association between changes in perceived neighborhood quality and concurrent, cross-sectional cognitive performance. An analytic sample of 592 Black adults (*M_age_* = 56.22; 58% female) recruited from 13 predetermined neighborhoods in Baltimore City, Maryland, from the ongoing prospective Healthy Aging in Neighborhoods of Diversity across the Life Span (HANDLS) study, was included. In the adjusted models, uncorrected multivariable regression indicated that increases in neighborhood disorder were associated with worse attention and visual orientation (*β* = 0.17, *p* < 0.001), while increases in social cohesion and social control were associated with better performance in several tasks relating to memory, attention, executive function/reasoning, and visual orientation (*βs* = −0.12–−0.10, *p* < 0.05). Interestingly, increasing social control was also associated with slower performance on a measure of executive functioning and reasoning (*β* = 0.12, *p* < 0.05). However, after controlling for multiple comparisons, only the association between neighborhood disorder and attention and visual orientation remained significant. Study findings serve as a springboard for future research linking changes in perceived neighborhood quality and concurrent, cross-sectional cognitive performance. These results may also lend support to the investment in neighborhoods as a pathway toward better understanding the association between neighborhood perception and cognitive performance, which may relate to reductions in cognitive decline and in the likelihood of dementia among Black adults who are at greatest risk for late-course diagnosis.

## 1. Introduction

The intersection of neighborhood characteristics and cognitive function serves as a public health opportunity. Recommendations from the National Institutes of Health [1,2] and the Office of Disease Prevention and Health Promotion [3] leverage the socioecological model to identify multilevel determinants (e.g., social and community context, neighborhood and built environment) relevant to our understanding of health and health disparities. The socioecological model highlights the interaction of a series of systems (i.e., microsystem, mesosystem, exosystem, macrosystem, and chronosystem) that influence an individual’s development (e.g., health outcomes) over time [4]. This is particularly important as an estimated 14 million individuals will be living with Alzheimer’s disease and related dementias (ADRD) by 2060, with a disproportionate number affecting individuals of Hispanic, African American/Black, and American Indian/Alaska Native racial/ethnic origins [5]. This projected increase in ADRD will have economic implications and place a burden on caregivers and on social and structural environments to support those living with ADRD. Using a systems-based lens that focuses on aspects of the environment as drivers of disparities in cognitive health outcomes may be beneficial. Thus, context-based factors provide an avenue for community-level or policy-based interventions that aim to identify and support healthy aging (e.g., cognitive function) in the population. 

In fact, Black adults have a disproportionately greater risk for an Alzheimer’s disease/Alzheimer’s disease and related dementias (AD/ADRD) diagnosis compared to White adults [6]. Black adults are also likely to receive dementia diagnoses later in the course of the disease compared to non-Hispanic White adults [6]. These observable AD/ADRD disparities may be partially attributed to performance on cognitive measures, which are commonly used as a clinical diagnostic indicator for AD/ADRD risk [7]. While the prior literature has identified pertinent biopsychosocial factors (e.g., hypertension, diabetes, and education) that contribute to Black adults’ risk for poor performance on cognitive measures or AD/ADRD risk [8], fewer studies have extended beyond individual-level factors and explored the extent to which contextual factors (e.g., neighborhood characteristics) enhance the understanding of AD/ADRD risk among Black adults, particularly by examining this association within Black only samples. Exploration of contextual factors offers an opportunity to gain a deeper understanding of the heterogeneous patterns of cognitive health among Black adults, as well as the individual behaviors and experiences (e.g., education and stress) that are linked to cognitive functioning [9]. 

A recent systematic review reported significant associations between neighborhood characteristics (i.e., perceived neighborhood disorder and social cohesion) and cognitive performance [10]. For example, in a cross-sectional study among midlife and older Hispanic/Latino adults, lower perceived physical disorder (e.g., excessive noise, trash and litter, and violence) was associated with higher global cognitive function and memory scores after adjusting for demographic (e.g., age, household income, education) and health covariates (e.g., depressive symptoms) [11]. Contrastingly, higher perceived social cohesion (e.g., trusting, friendliness, and assistance of neighbors) was associated with lower global cognition, verbal learning, verbal fluency, and processing speed [11]. While these significant associations were observable among women, they were not found among men. A study using Health and Retirement Study (HRS) data observed that higher perceived physical disorder was associated with worse performance on measures of episodic memory and verbal fluency at baseline. However, perceived neighborhood disorder at baseline was not associated with changes in performance on these cognitive measures [12]. Social cohesion was also associated with better episodic memory and verbal fluency at baseline. Additionally, social cohesion was associated with a slower rate of performance decline strictly for the verbal fluency measure, even after adjusting for covariates (e.g., age, education, and health conditions) [12]. A second study using HRS data provided supporting evidence that perceived neighborhood environment was associated with different cognitive domains among older adults. In particular, greater perceived physical disorder was related to poorer episodic memory, whereas greater perceived social cohesion was related to better semantic verbal fluency [13].

Existing empirical evidence suggests that adverse (e.g., neighborhood disorder) and protective (e.g., social cohesion) facets of the neighborhood may relate to cognitive function in opposing ways. However, much of the literature has neither strategically examined how changes in neighborhood perception may relate to cognition, nor examined the association between perceived neighborhood characteristics and cognitive performance among Black adults. In fact, a scoping review on neighborhoods and cognitive health disparities among middle-aged to older adults suggested that an increase in racially and ethnically represented research is needed to disentangle the association between neighborhood and specific cognitive function domains [14]. Examining the role of neighborhood context and its influence in shaping cognition among Black adults is of particular importance when placed against the socio-historical backdrop of the United States. A robust body of literature has reviewed how structural drivers have operated to influence health in adverse ways within the United States [15]. Specifically, racially and ethnically diverse communities have disproportionately faced the burden of residential segregation and discriminatory environmental practices (e.g., redlining, housing inequality) [16,17]. 

Moreover, given the geographical context of the current sample, these historical practices and policies have given rise to two Baltimores, also known as the Black Butterfly, in which Black residents live in communities in a butterfly-like shape on the east and west of Baltimore, where economic opportunities, investments, and resources are less abundant [18]. Reduced access to social, economic, and environmental opportunities is a driver of poorer health outcomes. However, the dynamic nature of neighborhood perception is far less examined, as many studies highlight the cross-sectional nature of neighborhood and health. In particular, geographers advocate the ebb and flow of people’s views of their neighborhood as an inherent characteristic between people and places [19]. Among older residents, perceptions of changing communities have social, financial, and mental health consequences [20]. In fact, recent research has highlighted the importance of perceived measures of neighborhood, such that subjective rather than objective measures of neighborhood were significantly associated with longitudinal changes in cognitive performance [21]. Thus, the current study examined changes in perceived neighborhood quality and concurrent, cross-sectional computerized cognitive performance within middle-aged to older Black adults. Specifically, it was hypothesized that decreases in neighborhood physical and social disorder and increases in social cohesion and social control would be associated with better cognitive performance. 

## 2. Methods

The Healthy Aging in Neighborhoods of Diversity across the Life Span (HANDLS) study is an ongoing prospective interdisciplinary study initiated in 2004, designed to understand the sources of persistent health disparities in overall longevity, cardiovascular disease, and cerebrovascular disease [22]. A fixed cohort of 3720 socioeconomically diverse Black (*n* = 2198) and White (*n* = 1522) adults from 13 predetermined neighborhoods was recruited in Baltimore City, Maryland. Data were included from waves 3 and 4 collected between 2009 and 2017. Participants were invited to visit a Medical Research Vehicle (MRV), where they received an array of physical health (e.g., a comprehensive physical examination), cognitive, mental health, and sociodemographic assessments. Eligible participants were aged 30–64, able to give informed consent, able to perform at least five measures, and had valid photo identification. Exclusion criteria included pregnancy and being within six months of active cancer treatment. The HANDLS protocol was approved by the Institutional Review Board at the National Institutes of Health. The de-identified data were accessed on 15 April 2022. 

### 2.1. Measures

*Neighborhood*. Following previous methodology [23] sum scores were calculated for each aspect of the neighborhood environment (i.e., physical and social disorder, social cohesion, and social control). The neighborhood questionnaire administered in the HANDLS study at wave 3 and wave 4 was derived from validated dimensions suggested by previous research [24,25]. Response options for the neighborhood perception measures were scaled on a 5-point (1—Never to 5—Very Often) and a 4-point (1—Never to 4—Often) Likert scale for waves 3 and 4, respectively. Participants were administered an 11-item neighborhood disorder scale (e.g., frequency of graffiti, crime, and loitering), a 5-item social cohesion scale (e.g., neighbors can be trusted), and a 3-item social control scale (e.g., taking action if a fight occurs). Response options were recoded to create comparable scores across the neighborhood disorder scales such that participants reporting “5—Very Often” were recoded as “4—Often” within wave 3. Additionally, a principal component factor analysis did not include one item from the social cohesion scale. All social control items and one item from social cohesion were reverse-coded so that higher scores reflected better standing on the scale. Contrastingly, higher scores for neighborhood disorder reflected worse neighborhood disorder. For full details on the neighborhood scales used in this study, see Supplementary Tables S1 and S2. 

*Cognitive Performance*. The Joggle Research Battery (Joggle Research, Inc., Seattle, WA, USA) is an interactive game-like experience that tracks performance across various cognitive tasks using an iPad. The cognitive tasks considered for this study were Motor Praxis (MPT; speed), Visual Object Learning (VOLT; attention and visual orientation), NBack (NBack; memory and attention), Abstract Matching (AM; executive functioning and reasoning), Line Orientation (LOT; visual orientation), Digit Symbol Substitution (DSST; attention, executive functioning, and reasoning), Balloon Analog Risk task (BART; executive functioning and reasoning), and Psychomotor Vigilance (PVT; speed and attention). The tasks were designed to measure a variety of cognitive domains, including speed, attention, visual learning memory, working memory, and executive function/reasoning. Scores for a given task assessed the number of correct responses/accuracy or mean reaction time (in milliseconds). Terminology and categorization of the Joggle tasks were adopted from Gamaldo and colleagues [26]. The Joggle Research Battery was administered during wave 4 of the study.

*Covariates*. Given the previously observed correlations between these covariates and cognitive performance, several sociodemographic and health covariates were included [11]. Age was measured in years. Given that education quality has been uniquely associated with cognitive performance within Black adults beyond education years [27], education quality was assessed by the reading subtest of the Wide Range Achievement Test (WRAT) [28]. Sex was defined as (0) male and (1) female. Poverty status was defined as (0) below poverty (<125%) and (1) above (≥125%) poverty using the 2004 United States Census Bureau poverty threshold based on income, size of family, and related children under 18 years. Depressive symptoms were measured using the Center for Epidemiological Studies Depression (CES-D) scale [29]. A moved variable was included to determine whether participants had moved since their last data collection. Moving was captured using a binary response system, i.e., (0) No and (1) Yes. A medical condition sum score was calculated using six self-reported medical diagnoses: heart attack, hypertension, diabetes, stroke, renal disease, and liver disease. These medical conditions were selected based on prior observed associations between these diseases and cognitive performance [11]. The diagnoses of the diseases were captured using a binary response system, i.e., (0) No and (1) Yes. 

### 2.2. Statistical Analysis

Descriptive statistics (i.e., mean, standard deviation, and percentage/frequency) were computed to explore the sample’s sociodemographic, health, neighborhood quality, and cognitive performance characteristics. A change score was estimated for each neighborhood variable in which the value at wave 3 (June 2009–July 2013) was subtracted from the value at wave 4 (September 2013–September 2017). Multivariable linear regression analyses were conducted, with each cognitive measure serving as the outcome variable. Models were adjusted for age, sex, reading literacy, poverty status, having moved, depressive symptoms, medical condition history, and wave 3 neighborhood perception. Linear regression models were conducted to examine whether changes in perceived neighborhood quality were significantly associated with concurrent, cross-sectional cognitive performance at wave 4. Initial sociodemographic recruitment indicators, sex, WRAT, and poverty status, wave 3 neighborhood perception, and wave 4 age, moved, depressive symptoms, and medical condition history were included in the models. All regression analyses were performed using SAS 9.4. Additionally, to control for multiple comparisons, the False Discovery Rate (FDR) SAS function was applied to the study findings. 

## 3. Results

The analytic sample for this study included a total of 592 Black adults with complete data (Supplementary Figure S1). For the initial sociodemographic recruitment indicators, the majority of the sample was female (58%; *n* = 344). The participant sample had reading literacy test scores ranging from 18 to 56 (*M* = 41.56, *SD* = 6.93), and 60% of the sample reported an above poverty status. Participants ranged in age from 36 to 76 years (*M* = 56.22, *SD* = 8.94) at wave 4. Participants reported lower levels of depressive symptoms (*M* = 12.77, *SD* = 10.00), and most of the sample reported 0 or 1 medical condition (*M* = 1.02, *SD* = 0.89). Fifty-six percent (56%) of the sample indicated they had not moved from wave 3 to wave 4. Participants reported decreases in neighborhood disorder (*M* = −2.36, *SD* = 8.18) and social control (*M* = −0.18, *SD* = 3.42) over time. Contrastingly, participants reported an increase in social cohesion (*M* = 0.38, *SD* = 3.76) over time (Table 1). Compared to the analytic sample, the attrition sample was older, included more females, had lower reading literacy scores, and included more individuals with a below poverty status at baseline (Supplementary Table S3).

### 3.1. Increasing Neighborhood Disorder Associated with Worse Cognitive Performance

In the adjusted models, living in a neighborhood with increasing neighborhood disorder was associated with slower performance only on the VOLT (attention and visual orientation; *β* = 0.17, *p* < 0.001 (uncorrected), *p* < 0.05 (corrected); Table 2). Specifically, participants reporting increases in neighborhood disorder over time, from wave 3 to wave 4, were more likely to have slower average reaction times on the VOLT as assessed at wave 4. Change in neighborhood disorder was not associated with correct response or accuracy scores across all cognitive measures (Table 3).

### 3.2. Increasing Social Cohesion Associated with Better Cognitive Performance

Increasing social cohesion was associated with faster cognitive performance on the VOLT (attention and visual orientation; *β* = −0.10, *p* < 0.05 (uncorrected), *p* > 0.05 (corrected)), LOT (visual orientation; *β* = −0.12, *p* < 0.05 (uncorrected), *p* > 0.05 (corrected)), and NBack (memory and attention; *β* = −0.10, *p* < 0.05 (uncorrected), *p* > 0.05 (corrected); Table 4). Participants reporting improvements in social cohesion over time, from wave 3 to wave 4, were more likely to have faster average reaction times on these tasks as assessed at wave 4. Change in neighborhood social cohesion was not associated with correct response or accuracy scores across all cognitive measures (Table 5). Multiple linear regression models examining the association between change in neighborhood social cohesion and cognitive average reaction time (AvgRT).

### 3.3. Increasing Social Control Associated with Better and Worse Cognitive Performance

Increasing social control was associated with faster performance on the VOLT (attention and visual orientation; *β* = −0.11, *p* < 0.05 (uncorrected), *p* > 0.05 (corrected)) and DSST (attention, executive functioning, and reasoning; *β* = −0.12, *p* < 0.01 (uncorrected), *p* > 0.05 (corrected); Table 6). Participants reporting an increase in neighborhood social control from wave 3 to wave 4 were more likely to have faster average reaction times on two tasks as assessed at wave 4. Interestingly, increasing social control was associated with slower performance on AM (executive functioning and reasoning; *β* = 0.12, *p* < 0.05 (uncorrected), *p* > 0.05 (corrected); Table 6). Change in neighborhood social control was not associated with correct response or accuracy scores across all cognitive measures (Table 7).

## 4. Discussion

The current study examined the relationship between changes in perceived neighborhood quality and concurrent, cross-sectional cognitive performance among Black participants in the HANDLS study. Neighborhood disorder, social cohesion, and social control were significantly associated with cognitive performance. Improved perceptions of neighborhood quality were associated with better cognitive performance (average reaction time to complete task), even adjusting for sociodemographic and health covariates. Across the three measures of neighborhood, varying dimensions of cognition were more readily associated. Measures of attention were significantly associated with all three measures of neighborhood perception, suggesting that neighborhood assessments engage attentional processes regardless of the protective or adverse conditions of the environment. Contrastingly, executive function and reasoning were only significantly associated with social control. Items from this measure assessed how readily residents were willing to react in response to an event in their neighborhood, suggesting that heightened or decreased alertness may engage executive functioning and reasoning processes that encourage individuals to evaluate current conditions and adjust responses. Likewise, a measure of memory was also only significantly associated with social cohesion. The strength of social community bonds may positively influence memory through a reduction in stressful conditions and thus the promotion of better cognitive function. Additionally, measures of visual orientation were related to neighborhood disorder and social cohesion and control in opposing ways. Neighborhood disorder may reduce visual clarity through an increase in cognitive load, while social cohesion and control may encourage visual clarity through a reduction in cognitive load. However, after applying the false discovery rate (FDR) correction to the study findings, only one significant association remained. Specifically, increases in neighborhood disorder were associated with slower performance on a measure of attention and visual orientation. Only one effect remaining may suggest that the study’s conditions (e.g., sample size, statistical power) were insufficient to detect effects, or that the effects may not exist within the study. Nonetheless, more research is needed to examine how changes in neighborhood perception relate to concurrent, cross-sectional computerized cognitive performance, in order to better understand the patterns of the underlying pathways. Additionally, following common effect size indices highlighted by Sullivan and Feinn [30], the magnitude of observed associations reflected a medium effect size for average reaction time on a cognitive test of attention and executive function/reasoning (Δ*R*^2^: 0.19). Smaller effect sizes were observed for average reaction time on cognitive tests of visual orientation, memory, attention, and executive functioning/reasoning (Δ*R*^2^s: 0.05–0.08). 

These findings may support the notion that Black adults’ perceptions of their neighborhood (physical or social characteristics) are not static but dynamic over time. However, studies examining changes in neighborhood perception and cognition among Black residents are limited, specifically among middle-aged and older adults. One study identified that increasing perceived neighborhood fear among emerging young adults was associated with increased depressive symptoms [31]. Largely, studies focus on the neighborhood perception-cognition association cross-sectionally. A study examining the role of intersectional identity (i.e., race/ethnicity, gender, and education) between neighborhood perception and cognitive functioning found Black adults with fewer years of education had better cognitive performance on the Telephone Interview Cognitive Screen (TICS) assessment even when residing in less favorable neighborhoods [32]. The current study’s findings expand upon this prior observation by illustrating that capturing changes in neighborhood perception may be meaningfully linked to performance, particularly for specific cognitive abilities in Black adults. 

Furthermore, applying an ecological model of cognitive health, which aims to explain how neighborhood contexts (i.e., physical and social) contribute to the cognitive functioning of aging adults [33], underscores the importance of our findings. Specifically, certain neighborhood conditions or characteristics may facilitate or constrain its residents’ ability to engage in health-promoting behaviors that can directly and indirectly affect cognition. For example, a study examining the positive and negative physical attributes of a neighborhood found that access to specific neighborhood features (e.g., recreation centers, civic and social organizations) contributed most significantly to differences in cognitive function across a sample of geographically diverse Black and White adults [34]. Likewise, prior research has emphasized the role of neighborhood features that support the physical activity of older adults [35], which can serve as a neuroprotective pathway through the reduction in stress, anxiety, inflammation, and operating through cardiovascular mechanisms which have been linked to cognitive function. Similarly, a body of literature emphasizes the role of social support and social engagement in the cognitive function of older adults [36]. Socially supportive networks or activities may serve as mechanisms that modulate the effects of stressful events that can be deleterious to health, or through the influence of behaviors (e.g., food, services, physical activity). Thus, neighborhoods that support social interaction, social support, and community building may also serve as avenues through which cognitive health may benefit.

Our study findings highlight the importance of examining both physical and social neighborhood characteristics as they relate to cognition. Prior research conducted a similar approach of separating neighborhood characteristics into these two categories when exploring these characteristics as they relate to cardiovascular health [37], a biomarker of cognitive health within Black adults [38]. For example, residents living in neighborhoods with better physical built characteristics (e.g., walkability scores, availability of healthy foods, and greater safety) and social cohesion were less likely to be hypertensive even after adjusting for age, sex, income, and education [37]. More recent research has observed that accessible physical built neighborhood characteristics (e.g., green space) are associated with a lower incidence of hypertension [39]. Future research is warranted to explore how other meaningful metrics of neighborhood (e.g., green space and walkability) observed in prior studies but not included in the current study may also be related to cognitive functioning in Black adults.

Overall, our study results are consistent with prior cross-sectional and longitudinal analytic techniques conducted to examine the neighborhood-cognition association [10]. However, previous studies have primarily employed tasks focused on global cognition and working memory to assess the relationship between neighborhood perception and cognitive performance. Significant findings focused on memory, executive function/reasoning, and visual orientation from the Joggle battery were consistent with prior results [40]. Specifically, greater perceived neighborhood social cohesion and lower perceived neighborhood disorder were associated with better performance on these tasks. Nonetheless, we must be mindful of the inconsistencies between the current study and prior studies regarding participant sociodemographics (e.g., race/ethnicity, sex/gender), modality of the cognitive assessments (e.g., paper-and-pencil, mobile devices), study designs (e.g., cross-sectional, longitudinal), and measures of neighborhood quality (e.g., neighborhood socioeconomic status, walkability, infrastructure), as these differences in study methodology may have implications for interpreting results. Analytic approaches can influence research results in such a way that variability in analytic choices can lead to variability in observed results [41].

### Limitations and Strengths

Although this study reveals interesting findings, a few study limitations should be noted. First, the results may not be generalizable to all Black adults, as the participants from this study sample were initially recruited from Baltimore City, Maryland. These adults may be experiencing neighborhood characteristics that are unique to the city or areas surrounding Baltimore, such as the history of structural segregation, disinvestment, and the disparity between White and Black communities in Baltimore, often referred to as the Black Butterfly [18]. Second, the study models did not account for neighborhood clustering, as participants were recruited from 13 neighborhoods throughout Baltimore, Maryland. Without accounting for this clustering, results may overestimate the significance of the study findings. Third, the study did not include objective measures of neighborhood quality (e.g., area deprivation index, green space, blue space, pollution), but should be examined in future studies. This is particularly relevant as a body of literature posits that community context (e.g., accessible parks, walkable destinations) above and beyond socioeconomic characteristics independently affects the functional health, cognitive function, and social integration of its residents [34]. Fourth, this study specifically examined the association with computerized cognitive performance. The relationship between a traditional psychometric battery or even ambulatory assessments of cognition may reveal different findings. For example, a study observed greater satisfaction among older Black adults and less testing anxiety following the completion of computer-based cognitive batteries (i.e., Joggle, CogState) than a traditional paper-and-pencil psychometric battery [26]. Fifth, a bidirectional effect cannot be assumed but should be considered. Specifically, this study does not account for whether changes in cognitive performance (e.g., decline) may be related to changes in the reporting or perception of neighborhood quality over time. Sixth, selection bias, including survivor bias and loss to follow-up, must be acknowledged, as this may impact the generalizability of the results. The findings of this study may be attributed to the relatively small sample size after accounting for data missingness. Lastly, changes in perceptions of neighborhood quality were only measured across two time points. The inclusion of more time points may have implications for our understanding of the frequency of changes in perception of neighborhood quality as well as factors (e.g., health, stress, social support) that relate to these changes in perceptions of neighborhood. Relatedly, data were collected over 10 years ago; however, the HANDLS study is ongoing, providing opportunities to replicate the analyses in later waves or include more data.

Nonetheless, our study also includes several strengths. First, our study leveraged a socioeconomically diverse middle-aged-to-older Black adult sample from the HANDLS study residing in various neighborhoods throughout Baltimore, MD. Given the lack of prior research focusing on the heterogeneity of health outcomes among Black adults, i.e., within-group analysis, this study serves as a template for more research to examine the cognitive health outcomes of aging Black adults. Second, this study included a cognitive battery that assessed multiple domains of cognition. Prior studies examining the link between neighborhood environments and cognition have primarily focused on domain-specific cognitive function (e.g., working memory, attention) and overall cognitive function (e.g., global cognition) [9]. Including multiple domains of cognition advances our understanding of how perceptions of neighborhood relate to cognitive functioning. Lastly, our study uniquely explores the way in which changes or shifts in neighborhood perception may relate to cognitive function. Prior research has focused on the longitudinal trajectories of cognition through the measurement of change, decline, or incidence of dementia [10].

## 5. Conclusions

Black adults are at disproportionate risk for experiencing adverse sociocontextual events (e.g., neighborhood redlining) across their life course, which are related to morbidity and early mortality [20]. While our uncorrected study findings support public health recommendations to explore the influence of sociocontextual factors, such as neighborhood characteristics, on cognitive functioning and AD/ADRD risk within Black adults, the corrected study findings suggest that additional research is warranted to examine these pathways and further confirm these patterns of association. Additional research may also uncover the pivotal role of environmentally designed interventions in sustaining cognitive functioning. Further, it may encourage the incorporation of standard clinical procedures, such as periodically inquiring with Black patients about their perceptions of their residential neighborhood, to gain a more comprehensive understanding of the factors that could be attributed to their health, as well as to share accessible and appropriately tailored resources. Lastly, our findings highlight new potential research directions to explore, such as (a) the potential coupling between changes in neighborhood perceptions and concurrent, cross-sectional cognitive functioning or AD/ADRD risk and (b) biological and psychological mechanisms (e.g., inflammation and stress) that may explain these associations. 

## Figures and Tables

**Table 1:** Neighborhood quality, cognitive performance, sociodemographic, and health characteristics of the analytic sample.

	Wave 3		Wave 4	
	Mean (SD)	Range	Mean (SD) or *n* (%)	Range
*Neighborhood*				
Physical and Social Disorder	27.66 (8.96)	11–44	25.30 (8.71)	11–44
Social Cohesion	13.25 (3.59)	4–20	13.63 (3.31)	4–20
Social Control	11.05 (3.21)	3–15	10.87 (3.11)	3–15
*Cognitive Performance*				
*Average Reaction Time (ms)*				
Abstract Matching	-	-	3768.81 (1646.79)	292–20,376
Ballon Analog Risk	-	-	1373.20 (740.72)	224–7949
Digit Symbol Substitution	-	-	1610.56 (509.55)	439–4854
Line Orientation	-	-	11,221.60 (4200.25)	614–47,165
Motor Praxis	-	-	898.24 (325.08)	479–3921
NBack	-	-	993.75 (164.49)	609–2534
Psychomotor Vigilance	-	-	663.71 (1288.64)	217–20,222
Visual Object Learning	-	-	3415.97 (1142.80)	831–9752
*Correct Responses/Accuracy*				
Abstract Matching	-	-	15.38 (1.99)	9–20
Balloon Analog Risk			621.56 (286.96)	19–1000
Digit Symbol Substitution	-	-	54.04 (13.43)	1–93
Line Orientation	-	-	2.78 (1.81)	0–9
NBack	-	-	34.63 (7.18)	17–58
Psychomotor Vigilance	-	-	42.07 (5.25)	8–47
Visual Object Learning	-	-	13.25 (2.06)	8–19
*Covariates*				
Age			56.22 (8.94)	36–76
* Sex	-	-	-	
Male	-	-	248 (41.89%)	-
Female	-	-	344 (58.11%)	-
* WRAT	-	-	41.56 (6.93)	18–56
* Poverty Status	-	-	-	-
Below	-	-	234 (39.53%)	-
Above	-	-	358 (60.47%)	-
Moved	-	-	-	-
No	-	-	332 (56.08%)	-
Yes	-	-	260 (43.92%)	-
Depressive Symptoms	-	-	12.77 (10.00)	0–49
Medical Condition	-	-	1.02 (0.89)	0–4

* Initial sociodemographic recruitment indicator.

**Table 2:** Multiple linear regression models examining the association between change in neighborhood disorder and cognitive average reaction time (AvgRT).

	Neighborhood Physical and Social Disorder							
Cognitive Performance (AvgRT)	*B* (Unstandardized)	95% CI	*β* (Standardized)	*SE*	*p* (Uncorrected)	*p* (Corrected)	*R* ^2^	Adjusted *R*^2^
VOLT	23.13	[10.00, 36.26]	0.17	6.69	0.0006 ***	0.0144 *	0.07	0.06
BART	−1.70	[−10.28, 6.87]	−0.02	4.37	0.6966	0.8497	0.06	0.05
DSST	4.87	[−0.57, 10.32]	0.08	2.77	0.0795	0.2385	0.20	0.19
LOT	16.91	[−31.13, 64.94]	0.03	24.46	0.4896	0.8041	0.08	0.07
PVT	−4.52	[−19.70, 10.66]	−0.03	7.73	0.5590	0.8041	0.03	0.01
AM	1.42	[−17.64, 20.48]	0.01	9.70	0.8838	0.9295	0.06	0.05
NBACK	0.36	[−1.52, 2.24]	0.02	0.96	0.7081	0.8497	0.08	0.07
MPT	1.18	[−2.33, 4.69]	0.03	1.79	0.5103	0.8041	0.18	0.17

Note. Model testing for neighborhood disorder and cognitive performance after adjusting for sociodemographic (i.e., age, sex, reading literacy, poverty status, having moved), health (depressive symptoms and medical condition sum score), and wave 3 neighborhood quality covariates. Corrections for multiple comparisons were made using the False Discovery Rate (FDR) function in SAS. * *p* < 0.05. *** *p* < 0.001.

**Table 3:** Multiple linear regression models examining the association between change in neighborhood disorder and cognitive correct responses/accuracy.

	Neighborhood Physical and Social Disorder							
Cognitive Performance (Correct Response/Accuracy)	*B* (Unstandardized)	95% CI	*β* (Standardized)	*SE*	*p* (Uncorrected)	*p* (Corrected)	*R* ^2^	Adjusted *R*^2^
VOLT	−0.01	[−0.04, 0.01]	−0.06	0.01	0.2156	0.6468	0.06	0.05
BART	1.45	[−1.92, 4.83]	0.04	1.72	0.3980	0.7051	0.03	0.02
DSST	−0.09	[−0.23, 0.04]	−0.06	0.07	0.1742	0.6097	0.27	0.26
LOT	−0.01	[−0.03, 0.01]	−0.04	0.01	0.4365	0.7051	0.11	0.10
PVT	−0.02	[−0.08, 0.04]	−0.04	0.03	0.4307	0.7051	0.08	0.07
AM	−0.005	[−0.03, 0.02]	−0.02	0.01	0.6889	0.7367	0.02	0.001
NBACK	−0.06	[−0.15, 0.02]	−0.07	0.04	0.1484	0.6097	0.02	0.01

Note. Model testing for neighborhood disorder and cognitive performance after adjusting for sociodemographic (i.e., age, sex, reading literacy, poverty status, having moved), health (depressive symptoms and medical condition sum score), and wave 3 neighborhood quality covariates. Corrections for multiple comparisons were made using the False Discovery Rate (FDR) function in SAS.

**Table 4:** Multiple linear regression models examining the association between change in neigh-borhood social cohesion and cognitive average reaction time (AvgRT).

	Neighborhood Social Cohesion							
Cognitive Performance (AvgRT)	*B* (Unstandardized)	95% CI	*β* (Standardized)	*SE*	*p* (Uncorrected)	*p* (Corrected)	*R* ^2^	Adjusted *R*^2^
VOLT	−31.58	[−61.82, −1.34]	−0.10	15.40	0.0407 *	0.1437	0.06	0.05
BART	1.37	[−18.25, 20.99]	0.01	9.99	0.8908	0.9295	0.06	0.05
DSST	−7.36	[−19.83, 5.10]	−0.05	6.35	0.2464	0.5376	0.20	0.19
LOT	−131.59	[−241.04, −22.13]	−0.12	55.73	0.0185 *	0.1110	0.09	0.08
PVT	−4.64	[−39.40, 30.13]	−0.01	17.70	0.7934	0.9067	0.03	0.01
AM	−11.59	[−55.33, 32.15]	−0.03	22.27	0.6031	0.8041	0.06	0.04
NBACK	−4.44	[−8.73, −0.16]	−0.10	2.18	0.0419 *	0.1437	0.09	0.08
MPT	0.13	[−7.90, 8.17]	0.002	4.09	0.9740	0.9740	0.18	0.17

Note. Model testing for neighborhood social cohesion and cognitive performance after adjusting for sociodemographic (i.e., age, sex, reading literacy, poverty status, having moved), health (depressive symptoms and medical condition sum score), and wave 3 neighborhood quality covariates. Corrections for multiple comparisons were made using the False Discovery Rate (FDR) function in SAS. * *p* < 0.05.

**Table 5:** Multiple linear regression models examining the association between change in neighborhood social cohesion and cognitive correct responses/accuracy.

	Neighborhood Social Cohesion							
Cognitive Performance (Correct Response/Accuracy)	*B* (Unstandardized)	95% CI	*β* (Standardized)	*SE*	*P* (Uncorrected)	*p* (Corrected)	*R* ^2^	Adjusted *R*^2^
VOLT	0.03	[−0.02, 0.08]	0.06	0.03	0.2701	0.6636	0.07	0.05
BART	−0.86	[−8.59, 6.86]	−0.01	3.93	0.8260	0.8260	0.03	0.01
DSST	0.28	[−0.03, 0.60]	0.08	0.16	0.0752	0.6097	0.28	0.27
LOT	−0.02	[−0.07, 0.03]	−0.04	0.02	0.4073	0.7051	0.11	0.09
PVT	0.04	[−0.10, 0.17]	0.03	0.07	0.5894	0.7270	0.08	0.07
AM	−0.04	[−0.10, 0.01]	−0.08	0.03	0.1150	0.6097	0.02	0.01
NBACK	−0.06	[−0.25, 0.14]	−0.03	0.10	0.5754	0.7270	0.02	0.003

Note. Model testing for neighborhood social cohesion and cognitive performance after adjusting for sociodemographic (i.e., age, sex, reading literacy, poverty status, having moved), health (depressive symptoms and medical condition sum score), and wave 3 neighborhood quality covariates. Corrections for multiple comparisons were made using the False Discovery Rate (FDR) function in SAS.

**Table 6:** Multiple linear regression models examining the association between change in neighborhood social control and cognitive average reaction time (AvgRT).

	Neighborhood Social Control							
Cognitive Performance (AvgRT)	*B* (Unstandardized)	95% CI	*β* (Standardized)	*SE*	*p* (Uncorrected)	*p* (Corrected)	*R* ^2^	Adjusted *R*^2^
VOLT	−35.40	[−67.79, −3.01]	−0.11	16.49	0.0322 *	0.1437	0.06	0.05
BART	−5.77	[−26.77, 15.23]	−0.03	10.69	0.5894	0.8041	0.06	0.05
DSST	−18.29	[−31.59, −5.00]	−0.12	6.77	0.0071 **	0.0852	0.20	0.19
LOT	96.56	[−20.92, 214.03]	0.08	59.82	0.1070	0.2853	0.09	0.07
PVT	−14.61	[−51.76, 22.53]	−0.04	18.91	0.4400	0.8041	0.03	0.01
AM	59.63	[13.13, 106.14]	0.12	23.68	0.0120 *	0.0960	0.07	0.05
NBACK	−1.44	[−6.04, 3.16]	−0.03	2.34	0.5392	0.8041	0.08	0.07
MPT	−6.17	[−14.75, 2.40]	−0.06	4.37	0.1580	0.3792	0.19	0.17

Note. Model testing for neighborhood social control and cognitive performance after adjusting for sociodemographic (i.e., age, sex, reading literacy, poverty status, having moved), health (depressive symptoms and medical condition sum score), and wave 3 neighborhood quality covariates. Corrections for multiple comparisons were made using the False Discovery Rate (FDR) function in SAS. * *p* < 0.05. ** *p* < 0.01.

**Table 7:** Multiple linear regression models examining the association between change in neighborhood social control and cognitive correct responses/accuracy.

	Neighborhood Social Control							
Cognitive Performance (Correct Response/Accuracy)	*B* (Unstandardized)	95% CI	*β* (Standardized)	*SE*	*P* (Uncorrected)	*p* (Corrected)	*R* ^2^	Adjusted *R*^2^
VOLT	0.03	[−0.03, 0.09]	0.05	0.03	0.2844	0.6636	0.06	0.05
BART	−2.27	[−10.54, 6.00]	−0.03	4.21	0.5901	0.7270	0.03	0.02
DSST	0.26	[−0.07, 0.60]	0.07	0.17	0.1211	0.6097	0.28	0.26
LOT	0.01	[−0.04, 0.06]	0.02	0.03	0.7016	0.7367	0.11	0.10
PVT	0.14	[−0.01, 0.28]	0.09	0.07	0.0638	0.6097	0.09	0.08
AM	−0.02	[−0.08, 0.04]	−0.03	0.03	0.4916	0.7270	0.02	0.001
NBACK	0.05	[−0.16, 0.26]	0.02	0.11	0.6231	0.7270	0.02	0.003

Note. Model testing for neighborhood social control and cognitive performance after adjusting for sociodemographic (i.e., age, sex, reading literacy, poverty status, having moved), health (depressive symptoms and medical condition sum score), and wave 3 neighborhood quality covariates. Corrections for multiple comparisons were made using the False Discovery Rate (FDR) function in SAS.

## Data Availability

Data are available to researchers after approval of a research proposal and after they have agreed to confidentiality as required by the National Institutes of Health Institutional Review Board. Policies and forms are available at: https://handls.nih.gov (accessed on 6 August 2026).

## Supplementary Materials

The following supporting information can be downloaded at: https://www.mdpi.com/article/10.3390/ijerph23081055/s1, Table S1: Neighborhood measures assessed in wave 3; Table S2: Neighborhood measures assessed in wave 4; Table S3: Group differences between attrition and analytic Black adult samples using wave 1 data; Figure S1: Flowchart illustrating reduction in sample size for analytic sample (*n* = 592).
