# Supplementary material for: Association Between Changes in Perceived Neighborhood and Cognitive Performance Within Healthy Aging in Neighborhoods of Diversity Across the Life Span (HANDLS) Black Adults
Source: Int J Environ Res Public Health. 2026 Aug 14;23(8):1055. doi: 10.3390/ijerph23081055 (PMC13513463; doi:10.3390/ijerph23081055)
Supplement: Supplementary file 1 [file ijerph-23-01055-s001.zip › ijerph-4375418-supplementary.pdf]

Table S1. Neighborhood measures assessed in wave 3.

**Neighborhood physical and social disorder**					
How often do you see…	*Never*	*Rarely*	*Sometimes*	*Often*	*Very Often*
graffiti on buildings and walls in your neighborhood?	1	2	3	4	5
litter on the sidewalks and streets in your neighborhood?	1	2	3	4	5
drug dealers, drug users or drunks hanging around in your neighborhood?	1	2	3	4	5
gang activity in your neighborhood?	1	2	3	4	5
disorderly or misbehaving groups of teens or children in your neighborhood?	1	2	3	4	5
prostitution in your neighborhood?	1	2	3	4	5
vacant, abandoned, or boarded up buildings in your neighborhood?	1	2	3	4	5
broken windows in your neighborhood?	1	2	3	4	5
How rare is serious crime such as assault, mugging, or robbery in your neighborhood?	1	2	3	4	5
houses or yards not kept up in your neighborhood?	1	2	3	4	5

**Neighborhood social cohesion**					
	Disagree strongly	*Disagree somewhat*	*Neither agree nor disagree*	*Agree somewhat*	*Agree Strongly*
People in my neighborhood are willing to help their neighbors.	1	2	3	4	5
I live in a close-knit neighborhood.	1	2	3	4	5
People in my neighborhood can be trusted.	1	2	3	4	5
People in my neighborhood generally do not get along with each other.	1	2	3	4	5

*People in my neighborhood do not share the same values.	1	2	3	4	5
**Neighborhood social control**					
	Very likely	*Likely*	*Neither likely nor unlikely*	*Unlikely*	*Very unlikely*
*If children were spray-painting graffiti on a local building, how likely would you say that your neighbors would take action?	1	2	3	4	5
*If children were showing disrespect to an adult, how likely would you say that your neighbors would take action?	1	2	3	4	5
*If a fight broke out in front of your house, how likely would you say that your neighbors would take action?	1	2	3	4	5

*Item reverse-coded for analyses.

Table S2. Neighborhood measures assessed in wave 4.

**Neighborhood physical and social disorder**				
How often do you see…	*Never*	*Rarely*	*Sometimes*	*Often*
graffiti on buildings and walls in your neighborhood?	1	2	3	4
litter on the sidewalks and streets in your neighborhood?	1	2	3	4
drug dealers, drug users or drunks hanging around in your neighborhood?	1	2	3	4
gang activity in your neighborhood?	1	2	3	4
disorderly or misbehaving groups of teens or children in your neighborhood?	1	2	3	4
prostitution in your neighborhood?	1	2	3	4
vacant, abandoned, or boarded up buildings in your neighborhood?	1	2	3	4
broken windows in your neighborhood?	1	2	3	4
How rare is serious crime such as assault, mugging, or robbery in your neighborhood?	1	2	3	4
houses or yards not kept up in your neighborhood?	1	2	3	4

**Neighborhood social cohesion**					
	Disagree strongly	*Disagree somewhat*	*Neither agree nor disagree*	*Agree somewhat*	*Agree Strongly*
People in my neighborhood are willing to help their neighbors.	1	2	3	4	5
I live in a close-knit neighborhood.	1	2	3	4	5
People in my neighborhood can be trusted.	1	2	3	4	5
People in my neighborhood generally do not get along with each other.	1	2	3	4	5

*People in my neighborhood do not share the same values.	1	2	3	4	5
**Neighborhood social control**					
	Very likely	*Likely*	*Neither likely nor unlikely*	*Unlikely*	*Very unlikely*
*If children were spray-painting graffiti on a local building, how likely would you say that your neighbors would take action?	1	2	3	4	5
*If children were showing disrespect to an adult, how likely would you say that your neighbors would take action?	1	2	3	4	5
*If a fight broke out in front of your house, how likely would you say that your neighbors would take action?	1	2	3	4	5

*Item reverse-coded for analyses.

Table S3. Group differences between attrition and analytic Black adult samples using wave 1 data.

	Attrition Black Adult Sample		Analytic Black Adult Sample		Group Differences
Wave 1	*n*	*Mean (SD)*	*n*	*Mean (SD)*	*p*
Age	1606	48.8.63 (9.48)	592	47.11 (8.78)	<.001
Sex					<.05
Male	749	-	248	-	
Female	857	-	344	-	
WRAT	1179	39.39 (8.19)		41.56 (6.93)	<.001
Poverty Status					<.001
Below	808	-	234	-	
Above	798	-	358	-	

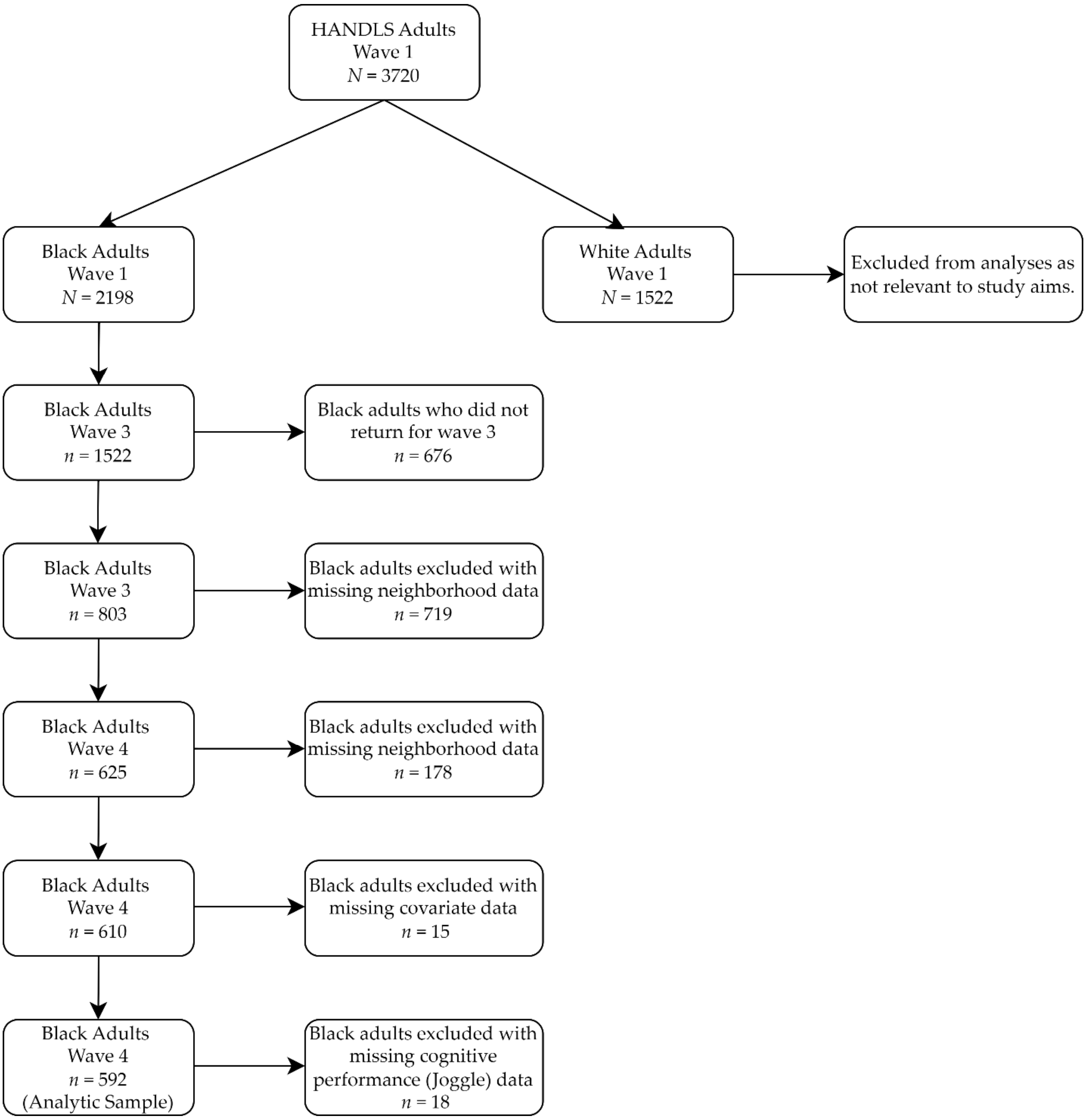

Figure S1. Flowchart illustrating reduction in sample size for analytic sample (n = 592).
